# Liraglutide improves sevoflurane-induced postoperative cognitive dysfunction via activating autophagy and inhibiting apoptosis

**DOI:** 10.18632/aging.205558

**Published:** 2024-02-15

**Authors:** Ying Hu, Haijin Huang, Yao Jiang, Jingling Zhang, Yang Zhang, Ying Tian, Qin Zhang

**Affiliations:** 1 Department of Endocrinology and Metabolism, The 1st Affiliated Hospital, Jiangxi Medical College, Nanchang University, Nanchang 330006, Jiangxi, China; 2 Jiangxi Clinical Research Center for Endocrine and Metabolic Disease, Nanchang 330006, Jiangxi, China; 3 Jiangxi Branch of National Clinical Research Center for Metabolic Disease, Nanchang 330006, Jiangxi, China; 4 Department of Anesthesiology and Operative medicine, Medical Center of Anesthesiology and Pain, The 1st Affiliated Hospital, Jiangxi Medical College, Nanchang University, Nanchang 330006, Jiangxi, China; 5 Department of Anesthesiology, Medical Center of Anesthesiology and Pain, The 1st Affiliated Hospital, Jiangxi Medical College, Nanchang University, Nanchang 330006, Jiangxi, China

**Keywords:** liraglutide, sevoflurane, POCD, AMPK, autophagy

## Abstract

Background: Postoperative cognitive dysfunction (POCD) is a common postoperative complication in elderly patients. Liraglutide (LRG) has high homology (97%) with natural glucagon like peptide-1, and it has been proved to be effective in some nervous system diseases. Whether LRG could regulate POCD has not been reported.

Methods: Sevoflurane (Sev) was used to simulate postoperative cognitive dysfunction (POCD) model. Morris water maze test was performed to evaluate the memory ability and neurological function of rats. Escape latency, swim distance, crossing platform times, average velocity, and targeting quadrant time were analyzed. The cell apoptosis, mRNA and protein expression were measured through flow cytometry, PCR, and western blotting, respectively.

Results: LRG significantly improved the memory ability and neurological function of Sev-treated rats, but 3-MA reversed the effects of LRG. LRG remarkably inhibited apoptosis but up-regulated autophagy related proteins both *in vivo* and *in vitro* levels. However, knocking down AMPK could markedly reverse the influence of LRG on apoptosis, autophagy, and cell apoptosis.

Conclusions: LRG induced autophagy activation can maintain cell homeostasis and promote cell survival by blocking the apoptotic pathway. LRG could improve Sev-induced POCD via activating autophagy, inhibiting apoptosis, and regulating AMPK/mTOR signaling pathway. This study provides a novel therapeutic strategy for the prevention and treatment of POCD.

## INTRODUCTION

Postoperative cognitive dysfunction (POCD) is a common postoperative complication of the patients [1]. POCD is more likely to occur in elderly patients after surgery, and the incidence of POCD in elderly patients over 65 years of age is 9%~46% [2, 3]. Its high incidence rate significantly affects the postoperative outcome and long-term quality of life of the elderly patients. It is believed that POCD is caused by many factors, including patient’s age, anesthetic drugs, anesthetic methods, types of surgery, duration of anesthetic surgery, etc., [4]. Anesthetics, especially general anesthetics, are generally considered as one of the important risk factors for POCD [5].

Sevoflurane (Sev), as the most commonly used inhalation anesthetics in clinical practice, has the advantages of rapid induction of anesthesia, rapid recovery, and little negative impact on the hemodynamics of liver and kidney function [6]. With the deepening of research, some studies have found that Sev exposure can cause neurocognitive dysfunction, especially in the elderly brain [7]. Studies have shown that Sev anesthesia can lead to the reduction of hippocampal neurogenesis and neuronal cell death, thus leading to cognitive dysfunction in elderly rats [8, 9]. Sev can cause neurocognitive dysfunction, which is more common in elderly patients. However, the regulatory mechanism of Sev in POCD remains unclear.

Glucagon like peptide-1 is an intestinal proinsulin active factor. Its comprehensive hypoglycemic effect makes it have great potential in the treatment of diabetes [10]. Liraglutide (LRG) has high homology (97%) with natural glucagon like peptide-1, and can directly act on the central nervous system through the blood brain barrier [11]. LRG has been proved to play a protective role in many kinds of nervous system diseases [12]. The regulatory function of LRG in Sev-induced POCD has not been fully explored.

Autophagy plays an important role in the growth, development and aging of the body [13]. As a way of programmed cell death, autophagy activation is considered to be a possible mechanism of neurotoxicity caused by general anesthesia drugs [14]. It was found that general anesthesia drugs led to morphological changes of mitochondria and synaptic conduction disorder in developing rat brain, accompanied by increased autophagy level [15]. The apoptosis in the POCD process has been reported before. Minocycline attenuated Sev-induced postoperative cognitive dysfunction in aged mice by suppressing hippocampal apoptosis [16]. If LRG could improve Sev-induced POCD through affecting autophagy or apoptosis remains unclear.

AMP activated protein kinase (AMPK) is an upstream molecule of mTOR. When cells are under stress including starvation and hypoxia, decreased ATP can activate AMPK, and activated AMPK regulates autophagy through a variety of signal pathways [17]. It was reported that ischemic preconditioning can provide neuroprotection by inducing AMPK dependent autophagy in rat ischemic stroke models [18]. In addition, LRG activated autophagy through AMPK/mTOR signaling pathway, reducing neuronal damage caused by diabetes [19]. The above research suggests that AMPK is not only an important node in the intracellular energy metabolism monitoring system, but also an important upstream signaling pathway for regulating autophagy.

In this study, elderly rats anesthetized with Sev were taken as experimental objects. Autophagy inhibitors and LRG were used to intervene animals, to evaluate neurological function, to detect the expression levels of autophagy and apoptosis related proteins. The role of autophagy in the pathogenesis of Sev-induced neurotoxicity was explored. In addition, the signaling pathway and molecular mechanism of AMPK regulation were investigated by knocking down AMPK. We firstly reported the balance regulation of apoptosis and autophagy by LRG in the Sev-induced POCD. This study provides some experimental basis for further understanding the pathogenesis of Sev-induced neurotoxicity and searching for therapeutic targets in clinical practice.

## MATERIALS AND METHODS

### Cell culture

Rat hippocampal neuron cell line (H19-7) purchased from ATCC (#CRL-2526^™^) was used in this research. DMEM high glucose complete medium (#MA0212, Meilune, China) containing 10% FBS (#FBS-S031219, Newzerum, New Zealand), 100 U/mL penicillin (Meilune, #MA0110), 100 μG/mL streptomycin (Meilune, #MA0113) was used to culture cells. The cells were cultured in an incubator at 37°C, containing 5% CO_2_ and saturated humidity. The cells were digested with 0.25% trypsin (Sigma, USA), and then centrifuged at 1000 r/min for 3 min. The supernatant was discarded and the precipitated cells were collected for different experiments.

### Sev treatment in H19-7 cells

The Sev-treated cell model was established as described previously [20]. The cells were put to Billups-Rothenburg chamber. Sev (4%), CO_2_ (5%), and air (91%) were injected to chamber at 5 L/min within 5 min. Then, the chamber was sealed for 6 h at 37°C. The cells in the group control and LRG were treated in normal culture environment without Sev induction. 100 nM LRG and 10 mM 3-MA were used to treat cells for 6 h, respectively.

### Flow cytometry

After digestion, cells were centrifuged at 1500 g for 8 min, and supernatant was discarded. Propidium iodide and Annexin V-FITC (#C1062L, Beyotime, Beijing, China) were used to incubate cells in the dark for 15 min. Flow cytometry was performed to analyse cell apoptosis.

### Western blotting

After sacrifice, the brains of rats were taken under low temperature and the hippocampus was isolated on ice. The left hippocampus was put into the cryopreservation tube and stored at −80°C. The tissues were homogenated and centrifuged first. Lysis buffer (RIPA: PMSF = 100:1) was used to further lysed tissues. After centrifugation at 12000 r/min for 15 min, the protein supernatant was isolated, and protein content was measured using Pierce^™^ 660 nm reagent (Thermo Fisher Scientific, USA). 10% SDS-PAGE was performed to isolate protein, and the target proteins were transferred to a PVDF membrane. TBST with 5% non-fat milk was applied to incubate membranes for blocking. Primary antibodies incubation was performed at 4°C overnight. Quantity One software was used to analyze the gray value of protein bands. The following antibodies were used in this study. Rabbit anti-Bcl-2 (1:1000, ab32124, Abcam, UK), Rabbit anti-Bax (1:1000, ab32503, Abcam) Rabbit anti-Cleaved caspase-3 (1:1000, ab32042, Abcam), Rabbit anti-LC3 (1:500, ab63817, Abcam), Rabbit anti-GAPDH (1:500, ab245355, Abcam), Rabbit anti-AMPK (1:500, ab32047, Abcam), Rabbit anti-p-AMPK (1:500, ab133448, Abcam), Rabbit anti-mTOR (1:1000, ab134903, Abcam), Rabbit anti-p-mTOR (1:1000, ab109268, Abcam), Rabbit anti-P62 (1:1000, ab109012, Abcam).

### Animal experiment of Sev-induced cognitive dysfunction

All animal experiments were approved by the Animal Care and Welfare Committee of The First Affiliated Hospital of Nanchang University (CDYFY-IACUC-202211QR029). 16-month-old rats purchased from Charles River were randomly divided into 5 groups after adaptive feeding for 1 week. The 5 groups were listed as follows: Control, Sev, LRG, Sev+LRG, and Sev+LRG+3-MA. The animals in the group Sev, Sev+LRG, and Sev+LRG+3-MA were anesthetized for 6 hours (3.4% Sev anesthesia), and other groups were given air instead of Sev during the experiment. The rats were fasting for 8 h and drinking for 4 h before anesthesia. After 3.4% Sev was filled in the animal anesthesia box, the rats were put into the anesthesia box and inhaled 3.4% Sev for 6 h. During the anesthesia period, the respiratory amplitude, frequency and vital signs of the rats were observed. The anesthesia depth was determined by the color of the nose tip and toe tip of the rats. The body temperature was maintained at 38 ± 1°C by monitoring the anal temperature. The rats in other groups continued to inhale air for 6 hours. After the anesthesia, the rats were monitored to be able to move freely before returning to the cage. The animals in the group Sev+LRG were injected intraperitoneally with LRG (25 nmol/kg) 30 min before anesthesia. LRG (25 nmol/kg) and 3-MA (15 mg/kg) were injected intraperitoneally 30 min before anesthesia to rats in the group Sev+LRG+3-MA. The rats in the group control and Sev were injected with same amount of PBS (#10010023, Gibco, USA).

### Morris water maze test

In a circular black paint swimming pool (180 cm in diameter and 50 cm in depth), 30 cm deep water was added into it, and then add non-toxic black ink into the water to make the water black. The water surface is divided into four quadrants, and a platform with a diameter of 10 cm is placed in the center of one of the quadrants, and the height of the platform is about 2 cm lower than the water surface. The test started at 9:00 am every day. The water temperature was controlled at 24 ± 1°C. The rats were put into the water facing the pool wall at a fixed point, and the time when the rats find the platform in the water, and let rats stay for 30 s. If the rats could not find the platform within 60 s, the rats shall be led to the platform and kept for 30 s. The rats were trained 4 times a day for 5 consecutive days. On the sixth day, escape latency, swim distance, crossing platform times, average velocity, and targeting quadrant time were recorded.

### Immunofluorescence staining

Dewaxing was performed firs as described below. The sections were heated at 65°C for 1 h in oven. Incubation with xylene (5 min), 100% ethanol (10 min), 95% ethanol (10 min), 70% ethanol (10 min), and distilled water (5 min) were performed. Endogenous peroxidase was removed through 3% hydrogen peroxide incubation (5 min). Anti-LC3B antibody (#ab239416, Abcam, UK) was used to incubate tissues for 12 h at 4°C. After washing with PBS, the tissues were incubated with second antibodies (3 h). Finally, DAB regents (#P0203, Beyotime, China) were used to incubate slides. A fluorescence microscope was used to observe the staining. The calculation of IHC staining was performed with ImageJ software.

### Cell transfection

AMPK short interfering RNA (AMPK siRNA) was designed and constructed by GenePharma (Shanghai, China). Cell transfection was performed with Lipofectamine 3000 (Invitrogen, USA). The dose of AMPK siRNA used in cell transfection was 25 nM, and proteins were extracted 48 h after cell transfection. The transient transfection was constructed.

### Cell proliferation test

Cells in logarithmic growth phase were inoculated into 12-well plate. When the cell density reached 60%, the cells were treated with different concentrations of LRG (0, 50, 75, 100, 125, 150 nM) for 6 h. Then, Sev was used to treat cells for 6 h. After incubation with CCK8 reagent (#C0038, Beyotime, China) for 30 min, cell proliferation ability was measured by detecting absorbance at 450 nm.

### Statistical analysis

The data were presented as mean ± standard deviation. SPSS22.0 was used for statistical analysis. ANOVA was used to analyze data among multiple groups. Inspection level α = 0.05, and *p* < 0.05 was believed to be statistical difference.

### Availability of data and material

The data and material used to support the findings of this study are included within the manuscript and supplementary files.

## RESULTS

### LRG significantly improved the memory ability and neurological function of rats

**Figure 1:**
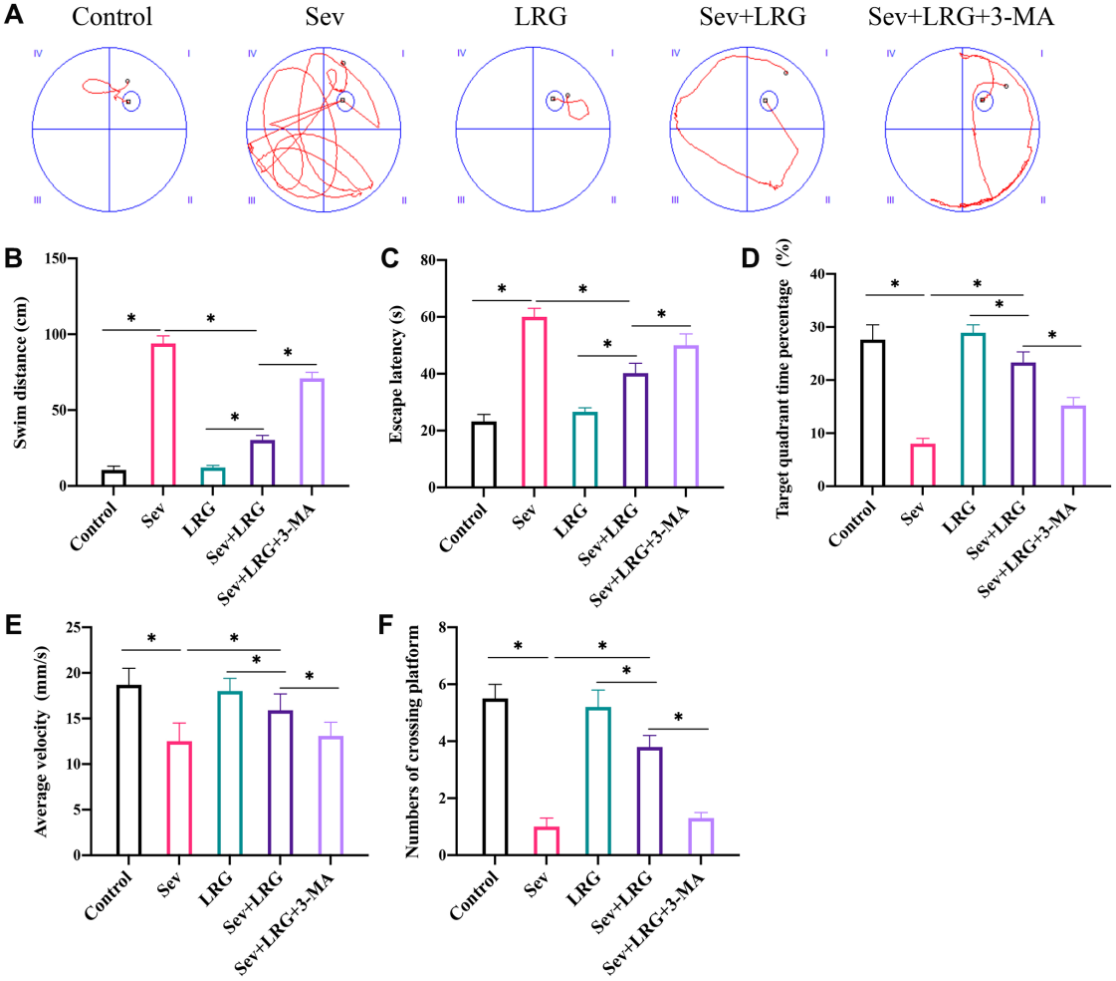
**LRG significantly improved the memory ability and neurological function of rats.** (**A**) Morris water maze test was performed to evaluate the influence of LRG on memory ability of rats; (**B**) Swim distance was calculated; (**C**) Escape latency was analyzed; (**D**) Target quadrant time percentage was analyzed; (**E**) Average velocity was recorded; (**F**) The number of crossing platform was calculated. ^*^p < 0.05.

Morris water maze test was performed to evaluate the influence of LRG and 3-MA on memory ability of animals. Significant increase of escape latency and swim distance were observed after Sev induction (Figure 1A–1C). However, LRG treatment significantly inhibited escape latency and swim distance compared with group Sev (Figure 1A–1C), and the change trends of escape latency and swim distance induced by LRG were inhibited by 3-MA. 3-MA is an inhibitor of autophagy. To further unfold the potential regulation of LRG on autophagy, 3-MA was added in this research. In addition, the decreased crossing platform times, average velocity, and targeting quadrant time after Sev induction were promoted by LRG treatment (Figure 1D–1F). However, the administration with 3-MA remarkably suppressed the effects of LRG.

### LRG remarkably inhibited apoptosis but up-regulated autophagy condition

**Figure 2:**
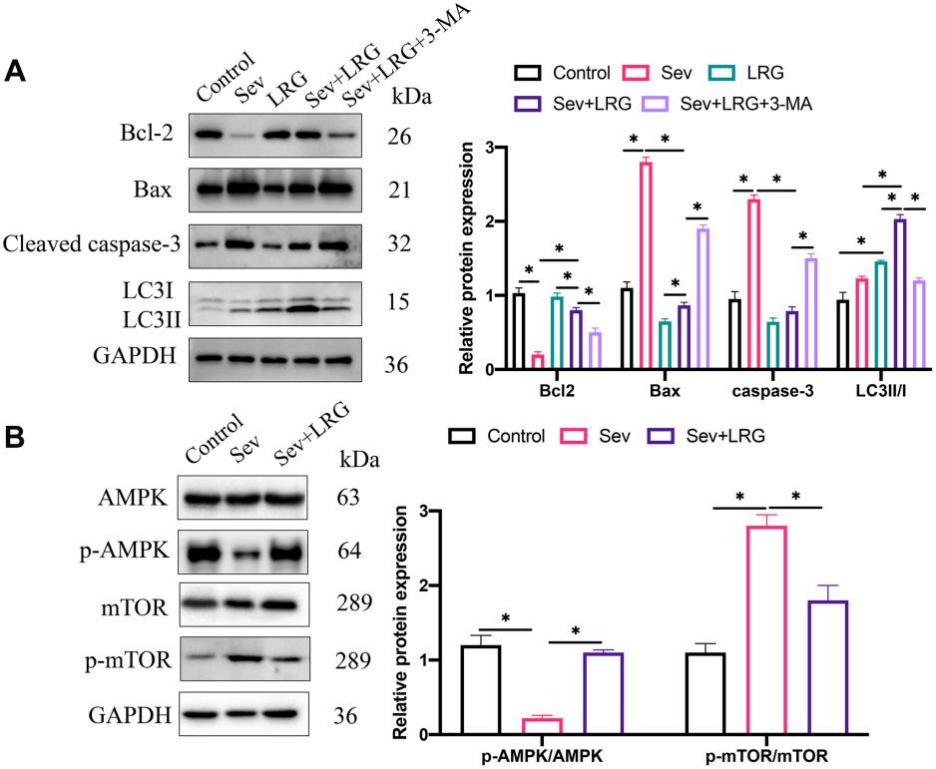
**LRG remarkably inhibited apoptosis but up-regulated autophagy condition.** (**A**) Apoptosis and autophagy related proteins were measured and analyzed; (**B**) The influence of LRG on AMPK/mTOR signaling pathway was analyzed ^*^p < 0.05.

The levels of apoptosis and autophagy related proteins expression have been believed to be closely linked with POCD. We found that pro-apoptotic proteins (Bax and cleaved caspase-3) were up-regulated, but Bcl-2 was inhibited after Sev induction (Figure 2A), suggesting that Sev remarkably increased the apoptosis intensity. However, LRG significantly suppressed the increased apoptosis level caused by Sev (Figure 2A), and 3-MA treatment remarkably promoted apoptosis. In addition, we found that LRG treatment markedly elevated LC3II/I ratio compared with group Sev, but the increased LC3II/I ratio caused by LRG was reversed by 3-MA (Figure 2A). We also investigated the influence of LRG on AMPK/mTOR signaling pathway. We found that treatment with LRG could significantly promote the expression level of p-AMPK/AMPK, but suppressed the level of p-mTOR/mTOR compared with group Sev (Figure 2B).

### The optimum concentration of LRG was investigated in cell level

**Figure 3:**
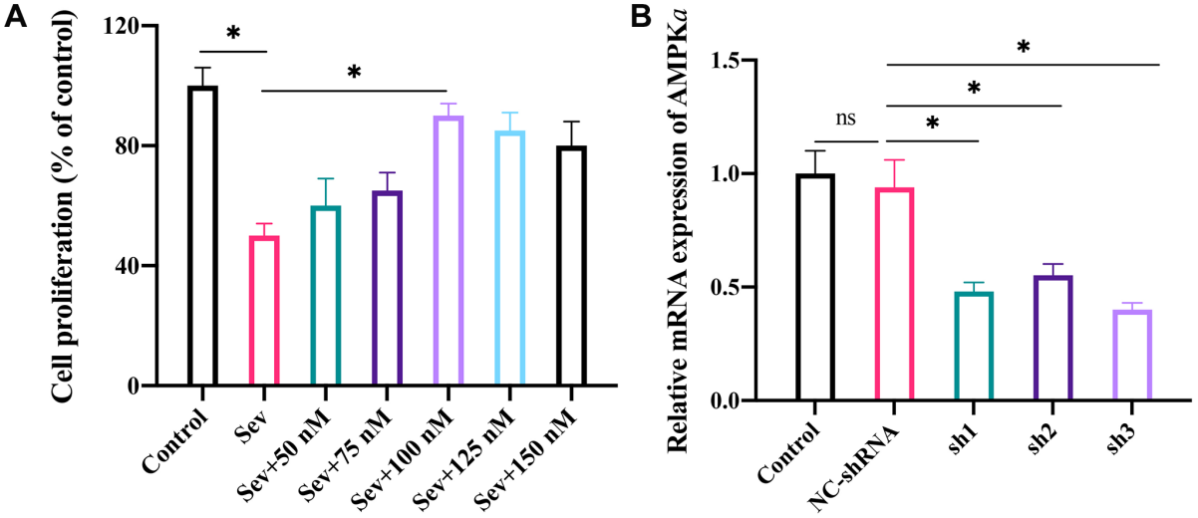
**The optimum concentration of LRG was investigated and the knockdown cell model of AMPK*a* was constructed in H19-7 cell line.** (**A**) The optimum concentration of LRG was investigated in cell level by incubating cells with different concentrations of LRG (0, 50, 75, 100, 125, and 150 nM); (**B**) Knockdown of AMPK*a* in cell level was constructed. ^*^p < 0.05.

The Sev-treated cell model was established first, and co-incubation with different concentrations of LRG (0, 50, 75, 100, 125, and 150 nM) was performed. We found that the cell proliferation ability was markedly restrained after Sev treatment (Figure 3A), but the cell viability was significantly increased after co-treatment with LRG (100 nM). In addition, with the increase of LRG concentration, the cell proliferation viability shown a slight weaker trend (Figure 3A). Therefore, 100 nM LRG was selected in the cell experiment. We also constructed sh-AMPK*a* in cell level (Figure 3B). After treatment with 3 designed shRNAs of AMPK*a*, the mRNA expression level of AMPK*a* was remarkably inhibited (Figure 3B), and shRNA-3 of AMPK*a* was chosen in the *in vitro* experiment.

### Cell apoptosis was suppressed and cell autophagy was promoted markedly by LRG in the Sev-treated cell model

**Figure 4:**
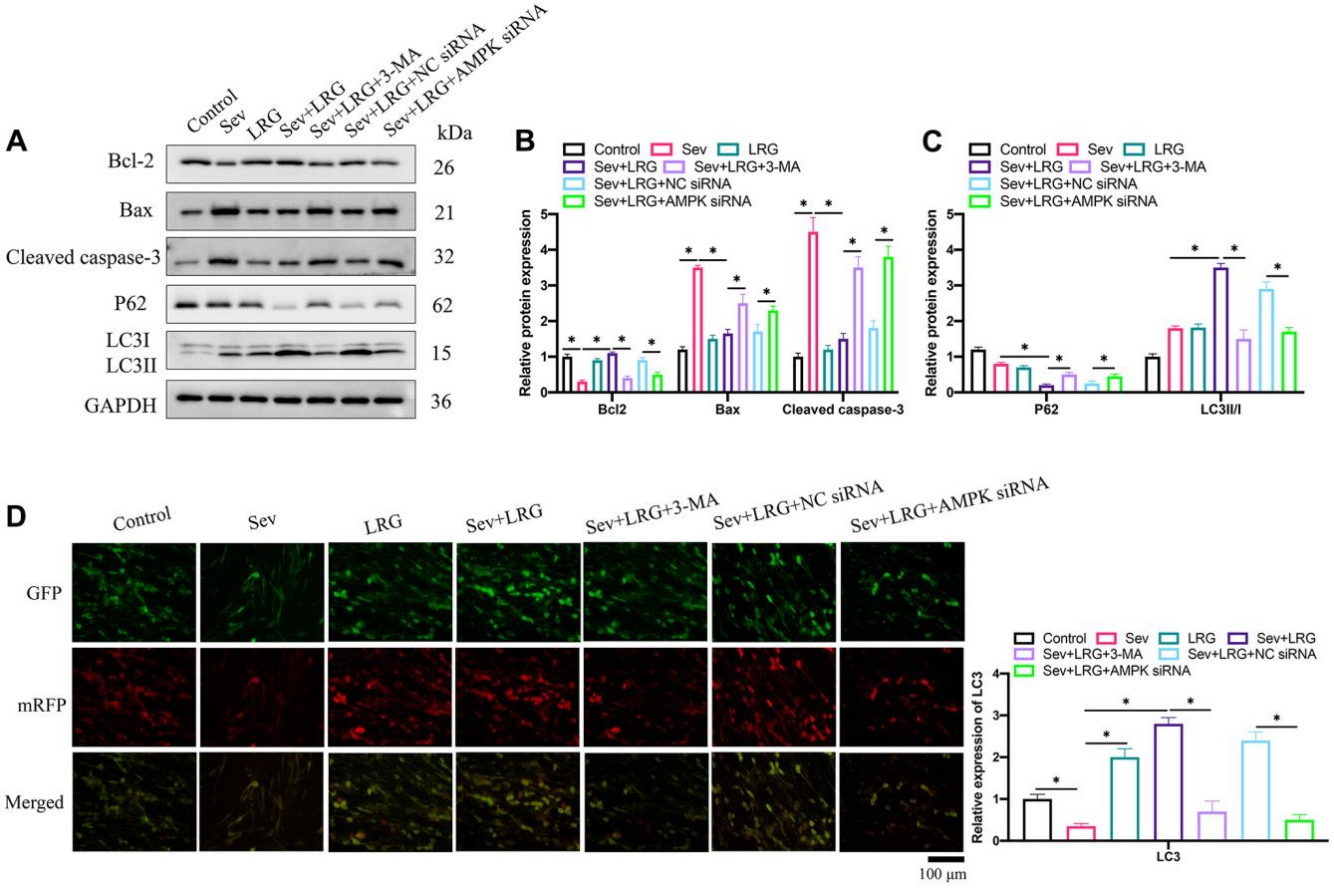
**Cell apoptosis was suppressed and cell autophagy was promoted markedly by LRG in the Sev-treated H19-7 cell line.** (**A**) Apoptosis and autophagy related proteins were measured with western blotting; (**B**) Apoptosis related proteins expression were analyzed; (**C**) Autophagy related proteins were analyzed; (**D**) The level of LC3 expression was investigated. ^*^p < 0.05.

Pro-apoptotic proteins (Bax and cleaved caspase-3) were up-regulated, but Bcl-2 was inhibited after Sev induction (Figure 4A, 4B). However, LRG significantly suppressed the increased apoptosis level caused by Sev (Figure 4A, 4B). In addition, LRG treatment markedly elevated LC3II/I ratio, but inhibited P62 compared with group Sev (Figure 4A, 4B). However, the influence of LRG on autophagy and apoptosis related proteins expression levels were reversed by AMPK siRNA (Figure 4A–4C). These findings indicate that AMPK might be involved in the regulation of LRG in autophagy and apoptosis. LC3 expression level was also investigated through measuring the immunofluorescence of mRFP (Figure 4D). Similar results to western blotting data were achieved (Figure 4D). LRG treatment remarkably increased the level of LC3 compared with group Sev, but both 3-MA and AMPK siRNA cold reversed the effect of LRG.

### The decreased cell apoptosis caused by LRG treatment was reversed by knocking down AMPK *in vitro*

**Figure 5:**
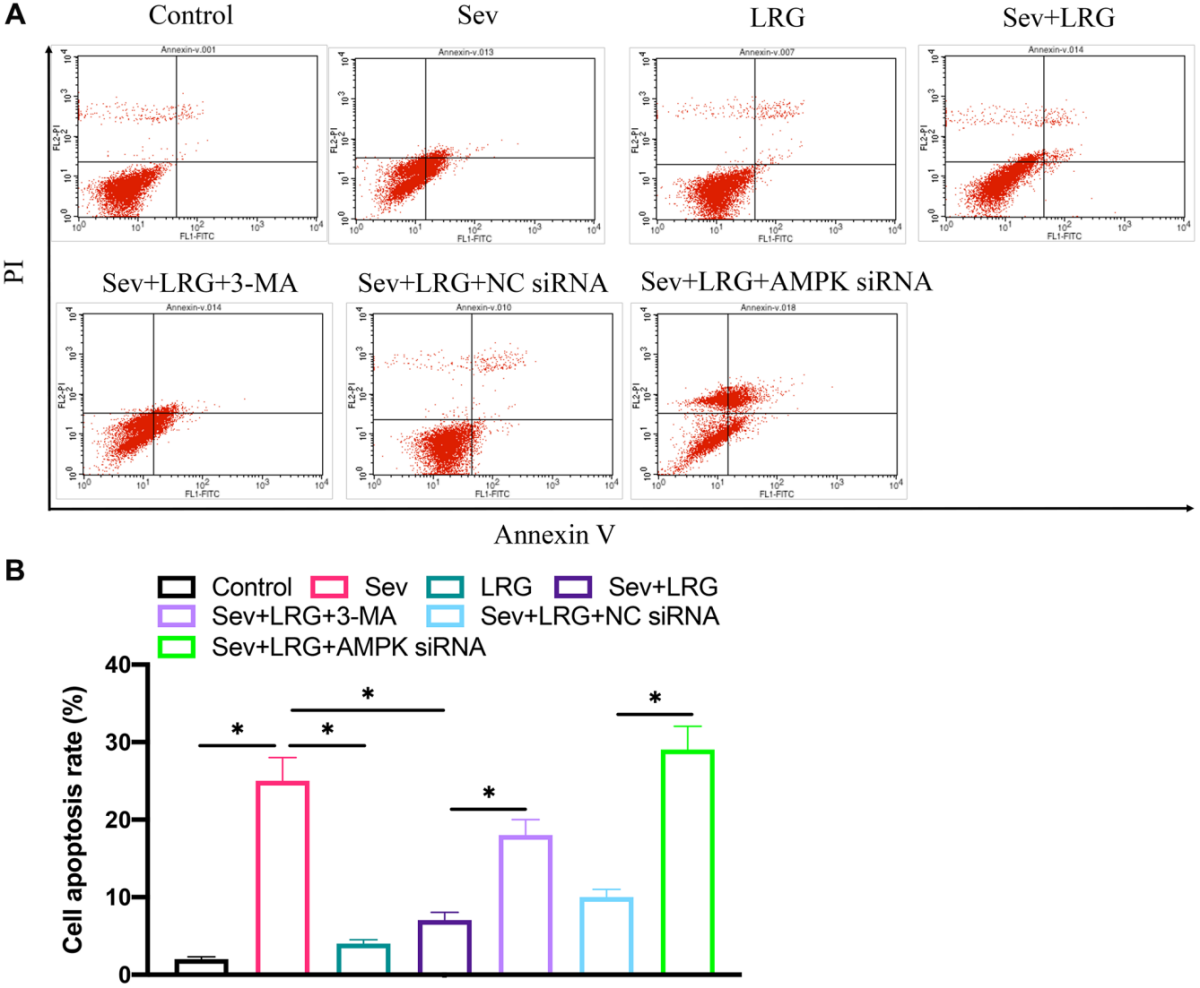
**Cell apoptosis was detected after LRG and AMPK siRNA treatment in H19-7 cell line.** (**A**) Cell apoptosis was detected after LRG and AMPK siRNA treatment; (**B**) Cell apoptosis was analyzed.

After administration of Sev, cell apoptosis was remarkably increased, but co-incubation with LRG could markedly inhibited the apoptosis level (Figure 5A, 5B). However, the supplement treatment with AMPK siRNA significantly promoted cell apoptosis level compared with group Sev+LRG+NC siRNA (Figure 5A, 5B).

## DISCUSSION

LRG could stimulate pancreas β cells to secrete glucose dependent insulin and inhibit glucagon secretion [21]. Meanwhile, LRG can penetrate the blood-brain barrier of rodents and plays an important role in regulating central nervous system diseases [22]. The study shows that LRG has protective effect on cerebral ischemia reperfusion injury in mice [23]. In addition, LRG improves nonalcoholic steatohepatitis by inhibiting the activation of NLRP3 inflammatory bodies and cell charring [24]. In this study, we further investigated the regulation of LRG on the balance of autophagy and apoptosis process during repair of postoperative cognitive dysfunction.

Autophagy is a lysosome dependent degradation pathway characterized by cytoplasmic vacuolar autophagy [25]. Autophagy could maintain the balance of energy and metabolism in cells through the degradation and reuse of damaged, denatured and aged proteins or organelles, and plays an important role in the growth, development and aging of the body [26]. Under normal physiological conditions, cells maintain a low basic autophagy level [27]. When cells are in hunger, stress, protein aggregation and other situations, autophagy level will be rapidly induced to increase, so as to maintain the normal metabolism of cells and the homeostasis of the internal environment [28]. Therefore, appropriate induction of autophagy can promote cells to better adapt to changes in the external environment. However, when autophagy continues to be abnormal, it may cause excessive autophagy of cells and lead to cell death.

The role of autophagy in regulating neurocognitive function is complicated. Studies have shown that Sev can increase cell apoptosis and reduce the proliferation of neural stem cells by activating autophagy, thus causing impairment of neurocognitive function [29, 30]. However, some studies suggest that autophagy is an important protective mechanism for cells to maintain homeostasis by clearing misfolded or aggregated proteins and damaged or aged organelles in cells [31]. Many studies show that autophagy plays a protective role in neurodegenerative diseases [32]. The occurrence and development of Alzheimer’s disease and Huntington’s syndrome are related to neuronal autophagy dysfunction [29]. In addition, Sev induced cognitive impairment in the elderly was related to impaired autophagy. In the present study, we found that LRG remarkably promoted the expression of autophagy related proteins, which might be the regulatory mechanisms improving neurological function.

Mammalian target of rapamycin (mTOR) plays a key role in autophagy induced signal transduction. mTOR can regulate cell growth, proliferation and autophagy [33]. AMPK activation can promote the phosphorylation of TSC2 protein, inhibit Rheb, and ultimately inhibit mTORCl activity, and up-regulate autophagy [34]. In this study, the increased autophagy condition induced by LRG was inhibited by AMPK siRNA. These findings indicate that LRG might exert effects through regulating AMPK signaling pathway.

## CONCLUSION

Sev anesthesia in aged rats can cause neuronal apoptosis, and cause cognitive dysfunction. Autophagy plays an important role in the occurrence and development of various neurodegenerative diseases and Sev-induced neurotoxicity. We demonstrated that LRG-induced autophagy activation can maintain cell homeostasis and promote cell survival by blocking the apoptotic pathway. This research might provide a novel therapeutic strategy for the prevention and treatment of POCD.

## References

[1] Lu J, Liu Z, Zhao Y, Liu X, He W, Zhang L. FGF19 improves sevoflurane-induced cognitive dysfunction in rats through the PGC-1α/BDNF/FNDC5 pathway. Tissue Cell. 2023; 81:102012. 10.1016/j.tice.2022.102012 36608639

[2] Qian X, Zheng S, Yu Y. CircUBE3B High Expression Participates in Sevoflurane-Induced Human Hippocampal Neuron Injury via Targeting miR-326 and Regulating MYD88 Expression. Neurotox Res. 2023; 41:16–28. 10.1007/s12640-022-00617-0 36585543

[3] Lin X, Chen Y, Zhang P, Chen G, Zhou Y, Yu X. The potential mechanism of postoperative cognitive dysfunction in older people. Exp Gerontol. 2020; 130:110791. 10.1016/j.exger.2019.110791 31765741

[4] Xie Y, Huang J, Chen Y. Exogenous recombinant Hsp70 attenuates sevoflurane anesthesia-induced cognitive dysfunction in aged mice. Brain Behav. 2023; 13:e2861. 10.1002/brb3.2861 36573756 PMC9847620

[5] Sun Y, Wang Y, Ye F, Cui V, Lin D, Shi H, Zhang Y, Wu A, Wei C. SIRT1 activation attenuates microglia-mediated synaptic engulfment in postoperative cognitive dysfunction. Front Aging Neurosci. 2022; 14:943842. 10.3389/fnagi.2022.943842 36437988 PMC9685341

[6] Lv G, Wang W, Sun M, Wang F, Ma Y, Li C. Inhibiting specificity protein 1 attenuated sevoflurane-induced mitochondrial stress and promoted autophagy in hippocampal neurons through PI3K/Akt/mTOR and α7-nAChR signaling. Neurosci Lett. 2023; 794:136995. 10.1016/j.neulet.2022.136995 36464148

[7] Duan GY, Duan ZX, Chen H, Chen F, Chen F, Du ZY, Chen LY, Lu KZ, Zuo ZY, Li H. Cognitive function and delirium following sevoflurane or propofol anesthesia for valve replacement surgery: A multicenter randomized controlled trial. Kaohsiung J Med Sci. 2023; 39:166–74. 10.1002/kjm2.12618 36354206 PMC11895883

[8] Wei FS, Rao MW, Huang YL, Chen SB, Wu YQ, Yang L. miR-182-5p Delivered by Plasma Exosomes Promotes Sevoflurane-Induced Neuroinflammation and Cognitive Dysfunction in Aged Rats with Postoperative Cognitive Dysfunction by Targeting Brain-Derived Neurotrophic Factor and Activating NF-κB Pathway. Neurotox Res. 2022; 40:1902–12. 10.1007/s12640-022-00597-1 36308704

[9] Yang J, Huang Q, Cao R, Cui Y. Effects of propofol and inhaled anesthetics on postoperative complications for the patients undergoing one lung ventilation: A meta-analysis. PLoS One. 2022; 17:e0266988. 10.1371/journal.pone.0266988 36264981 PMC9584365

[10] Lundgren JR, Janus C, Jensen SBK, Juhl CR, Olsen LM, Christensen RM, Svane MS, Bandholm T, Bojsen-Møller KN, Blond MB, Jensen JB, Stallknecht BM, Holst JJ, et al. Healthy Weight Loss Maintenance with Exercise, Liraglutide, or Both Combined. N Engl J Med. 2021; 384:1719–30. 10.1056/NEJMoa2028198 33951361

[11] O'Neil PM, Birkenfeld AL, McGowan B, Mosenzon O, Pedersen SD, Wharton S, Carson CG, Jepsen CH, Kabisch M, Wilding JPH. Efficacy and safety of semaglutide compared with liraglutide and placebo for weight loss in patients with obesity: a randomised, double-blind, placebo and active controlled, dose-ranging, phase 2 trial. Lancet. 2018; 392:637–49. 10.1016/S0140-6736(18)31773-2 30122305

[12] Wiciński M, Socha M, Malinowski B, Wódkiewicz E, Walczak M, Górski K, Słupski M, Pawlak-Osińska K. Liraglutide and its Neuroprotective Properties-Focus on Possible Biochemical Mechanisms in Alzheimer's Disease and Cerebral Ischemic Events. Int J Mol Sci. 2019; 20:1050. 10.3390/ijms20051050 30823403 PMC6429395

[13] Li S, Zhou Y, Hu H, Wang X, Xu J, Bai C, Yuan J, Zhang D. SIRT3 Enhances the Protective Role of Propofol in Postoperative Cognitive Dysfunction via Activating Autophagy Mediated by AMPK/mTOR Pathway. Front Biosci (Landmark Ed). 2022; 27:303. 10.31083/j.fbl2711303 36472103

[14] Li PJ, Guo YQ, Ding PY, Liu RB, Deng F, Feng XX, Yan WJ. Neuroprotective effects of a Smoothened receptor agonist against postoperative cognitive dysfunction by promoting autophagy in the dentate gyrus of aged rats. Neurol Res. 2019; 41:867–74. 10.1080/01616412.2019.1628411 31221056

[15] Yang N, Li L, Li Z, Ni C, Cao Y, Liu T, Tian M, Chui D, Guo X. Protective effect of dapsone on cognitive impairment induced by propofol involves hippocampal autophagy. Neurosci Lett. 2017; 649:85–92. 10.1016/j.neulet.2017.04.019 28411068

[16] Liang J, Han S, Ye C, Zhu H, Wu J, Nie Y, Chai G, Zhao P, Zhang D. Minocycline Attenuates Sevoflurane-Induced Postoperative Cognitive Dysfunction in Aged Mice by Suppressing Hippocampal Apoptosis and the Notch Signaling Pathway-Mediated Neuroinflammation. Brain Sci. 2023; 13:512. 10.3390/brainsci13030512 36979321 PMC10046414

[17] Shen B, Wang Y, Cheng J, Peng Y, Zhang Q, Li Z, Zhao L, Deng X, Feng H. Pterostilbene alleviated NAFLD via AMPK/mTOR signaling pathways and autophagy by promoting Nrf2. Phytomedicine. 2023; 109:154561. 10.1016/j.phymed.2022.154561 36610156

[18] Jiang T, Yu JT, Zhu XC, Zhang QQ, Tan MS, Cao L, Wang HF, Shi JQ, Gao L, Qin H, Zhang YD, Tan L. Ischemic preconditioning provides neuroprotection by induction of AMP-activated protein kinase-dependent autophagy in a rat model of ischemic stroke. Mol Neurobiol. 2015; 51:220–9. 10.1007/s12035-014-8725-6 24809692

[19] He Q, Sha S, Sun L, Zhang J, Dong M. GLP-1 analogue improves hepatic lipid accumulation by inducing autophagy via AMPK/mTOR pathway. Biochem Biophys Res Commun. 2016; 476:196–203. 10.1016/j.bbrc.2016.05.086 27208776

[20] Wu Z, Tan J, Lin L, Zhang W, Yuan W. microRNA-140-3p protects hippocampal neuron against pyroptosis to attenuate sevoflurane inhalation-induced post-operative cognitive dysfunction in rats via activation of HTR2A/ERK/Nrf2 axis by targeting DNMT1. Cell Death Discov. 2022; 8:290. 10.1038/s41420-022-01068-4 35710537 PMC9203584

[21] Erdogan MA, Erdogan A, Erbas O. The Anti-Seizure Effect of Liraglutide on Ptz-Induced Convulsions Through its Anti-Oxidant and Anti-Inflammatory Properties. Neurochem Res. 2023; 48:188–95. 10.1007/s11064-022-03736-4 36040609

[22] Qi L, Gao R, Chen Z, Lin D, Liu Z, Wang L, Lin L, Liu X, Liu X, Liu L. Liraglutide reduces oxidative stress and improves energy metabolism in methylglyoxal-induced SH-SY5Y cells. Neurotoxicology. 2022; 92:166–79. 10.1016/j.neuro.2022.08.007 35985417

[23] Zhao Y, Yu J, Ping F, Xu L, Li W, Zhang H, Li Y. Insulin and liraglutide attenuate brain pathology in diabetic mice by enhancing the Wnt/β-catenin signaling pathway. Exp Ther Med. 2022; 24:439. 10.3892/etm.2022.11366 35720633 PMC9185805

[24] Manavi MA. Neuroprotective effects of glucagon-like peptide-1 (GLP-1) analogues in epilepsy and associated comorbidities. Neuropeptides. 2022; 94:102250. 10.1016/j.npep.2022.102250 35561568

[25] Levine B, Kroemer G. Biological Functions of Autophagy Genes: A Disease Perspective. Cell. 2019; 176:11–42. 10.1016/j.cell.2018.09.048 30633901 PMC6347410

[26] D'Arcy MS. Cell death: a review of the major forms of apoptosis, necrosis and autophagy. Cell Biol Int. 2019; 43:582–92. 10.1002/cbin.11137 30958602

[27] Mizushima N, Levine B. Autophagy in Human Diseases. N Engl J Med. 2020; 383:1564–76. 10.1056/NEJMra2022774 33053285

[28] Klionsky DJ, Petroni G, Amaravadi RK, Baehrecke EH, Ballabio A, Boya P, Bravo-San Pedro JM, Cadwell K, Cecconi F, Choi AMK, Choi ME, Chu CT, Codogno P, et al. Autophagy in major human diseases. EMBO J. 2021; 40:e108863. 10.15252/embj.2021108863 34459017 PMC8488577

[29] Jinpiao Z, Zongze Z, Qiuyue Y, Peng F, Qi Z, Yanlin W, Chang C. Metformin attenuates sevoflurane-induced neurocognitive impairment through AMPK-ULK1-dependent autophagy in aged mice. Brain Res Bull. 2020; 157:18–25. 10.1016/j.brainresbull.2020.01.018 32014566

[30] Wang X, Dong Y, Zhang Y, Li T, Xie Z. Sevoflurane induces cognitive impairment in young mice via autophagy. PLoS One. 2019; 14:e0216372. 10.1371/journal.pone.0216372 31107909 PMC6527218

[31] Chen X, Zhang T, Zhang Y. Endoplasmic reticulum stress and autophagy in HIV-1-associated neurocognitive disorders. J Neurovirol. 2020; 26:824–33. 10.1007/s13365-020-00906-4 32918163

[32] Gu M, Mei XL, Zhao YN. Sepsis and Cerebral Dysfunction: BBB Damage, Neuroinflammation, Oxidative Stress, Apoptosis and Autophagy as Key Mediators and the Potential Therapeutic Approaches. Neurotox Res. 2021; 39:489–503. 10.1007/s12640-020-00270-5 32876918

[33] Jiang C, Zhao X, Jeong T, Kang JY, Park JH, Kim IS, Kim HS. Novel Specific Pyruvate Kinase M2 Inhibitor, Compound 3h, Induces Apoptosis and Autophagy through Suppressing Akt/mTOR Signaling Pathway in LNCaP Cells. Cancers (Basel). 2022; 15:265. 10.3390/cancers15010265 36612260 PMC9818605

[34] Qu N, Qu J, Huang N, Zhang K, Ye T, Shi J, Chen B, Kan C, Zhang J, Han F, Hou N, Sun X, Pan R. Calycosin induces autophagy and apoptosis via Sestrin2/AMPK/ mTOR in human papillary thyroid cancer cells. Front Pharmacol. 2022; 13:1056687. 10.3389/fphar.2022.1056687 36588732 PMC9800829

